# Dataset supporting the proteomic differences found between excretion/secretion products from two isolates of *Fasciola hepatica* newly excysted juveniles (NEJ) derived from different snail hosts

**DOI:** 10.1016/j.dib.2019.104272

**Published:** 2019-07-15

**Authors:** Lucía Sánchez Di Maggio, Lucas Tirloni, Antônio F.M. Pinto, Jolene K. Diedrich, John R. Yates, Carlos Carmona, Patricia Berasain, Itabajara da Silva Vaz

**Affiliations:** a Centro de Biotecnologia, Universidade Federal do Rio Grande do Sul, Porto Alegre, RS, Brazil; b Unidad de Biología Parasitaria, Facultad de Ciencias, Universidad de la República Oriental del Uruguay, Montevideo, Uruguay; c College of Veterinary Medicine, Department of Veterinary Pathobiology, Texas A&M University, College Station, TX, USA; d Department of Molecular Medicine, The Scripps Research Institute, CA, USA; e Faculdade de Veterinária, Universidade Federal do Rio Grande do Sul, Porto Alegre, RS, Brazil

**Keywords:** *Fasciola hepatica*, Intermediate host, Parasite-host interaction, Secretome

## Abstract

Here we present the proteomic profile datasets of two *Fasciola hepatica* NEJ isolates derived from different snail hosts: *Lymnaea viatrix* and *Pseudosuccinea columella*. The data used in the analysis are related to the article ‘A proteomic comparison of excretion/secretion products in *Fasciola hepatica* newly excysted juveniles (NEJ) derived from *Lymnaea viatrix* or *Pseudosuccinea columella*’ (Di Maggio et al., 2019)

**Table:** Specifications table

Subject area	Biology
More specific subject area	Parasite
Type of data	Table, excel files
How data was acquired	Easy NanoLC II coupled to a Q Exactive mass spectrometer (Thermo Fisher Scientific, USA) Integrated Proteomics Pipeline–IP2 (Integrated Proteomics Applications, http://www.integratedproteomics.com). *Fasciola hepatica* genome database [2] concatenated with *a Bos taurus* Uniprot reference database.
Data format	Raw and analyzed output data
Experimental factors	*F. hepatica* NEJ were excysted and cultured
Experimental features	*F. hepatica* NEJ were cultured and the excretion/secretion products were processed for mass spectrometry-based proteomic analysis
Data source location	Montevideo, Uruguay and Oregon, USA.
Data accessibility	Tables are available in this article. Raw files and search results have been deposited to Repository name: ProteomeXchange Consortium via the PRIDE partner repository [3]. Data identification number: PXD011991. Direct URL to data: http://proteomecentral.proteomexchange.org/cgi/GetDataset?ID=PXD011991 Analyzed data is provided along with this article as excel sheets
Related research article	Di Maggio, L.S., Tirloni, L., Pinto, A.F., Diedrich, J.K., Yates Iii, J.R., Carmona, C., Berasain. P., da Silva Vaz, I. A proteomic comparison of excretion/secretion products in *Fasciola hepatica* newly excysted juveniles (NEJ) derived from *Lymnaea viatrix* or *Pseudosuccinea columella*. Exp Parasitol. (2019) 1461–1475. https://doi.org/10.1016/j.exppara.2019.04.004[1]

**Table:** 

**Value of the data** • The data define the proteome of excretion/secretion products from two isolates of *Fasciola hepatica*. • The data provide experimental evidence at the level of protein expression for each isolate. • The data identify differences and similarities between the *F. hepatica* isolates. • The data provide a platform for further experiments to understand the mechanisms involved in the selection of biological material for vaccine trials

## 1 Data

Here we report the proteomic analysis of excretion/secretion products (ESP) from two isolates of *Fasciola hepatica* NEJ (Rubino strain and US Pacific North West wild strain), contrasting their similarities and differences in protein composition and abundance. The complete list of identified proteins and their corresponding peptides have been provided in Supplementary Table 1 (*F. hepatica*-derived proteins) and Supplementary Table 2 (*B. taurus*-derived proteins). Also, provide a comparison between *Bos taurus*-derived proteins identified in *Pseudosuccinea columella* and *Lymnea viatrix* NEJ ESP samples (Table 1).

**Table 1:** *Bos taurus*-derived proteins identified by LC-MS/MS in *Pseudosuccinea columella* and *Lymnea viatrix* NEJ ESP samples.

Accession number	Description	*L.viatrix*	*L. columella*
sp|A1L595|K1C17_BOVIN	Keratin, type II cytoskeletal 80		*
sp|P08728|K1C19_BOVIN	Keratin, type I cytoskeletal 19		*
tr|E1BGJ5|E1BGJ5_BOVIN	Complement component C1q receptor		*
tr|E1BJB1|E1BJB1_BOVIN	Tubulin		*
tr|F1MC11|F1MC11_BOVIN	Keratin		*
tr|F1MIH7|F1MIH7_BOVIN	Small GTPase mediated signal transduction		*
sp|P68138|ACTS_BOVIN	Actin, alpha skeletal muscle	*	
sp|Q5XQN5|K2C5_BOVIN	Keratin, type II cytoskeletal 5	*	
sp|P00760|TRY1_BOVIN	Cationic trypsin	*	*
sp|P06394|K1C10_BOVIN	Keratin	*	*
sp|P0CH28|UBC_BOVIN	Polyubiquitin-C	*	*
sp|Q29S21|K2C7_BOVIN	Keratin type II cytoskeletal 7	*	*
tr|F1MFW9|F1MFW9_BOVIN	Keratin	*	*
tr|F1MUY2|F1MUY2_BOVIN	Keratin	*	*
tr|F2Z4I6|F2Z4I6_BOVIN	Histone H2A	*	*
tr|G3N0V2|G3N0V2_BOVIN	Keratin	*	*
tr|M0QVY0|M0QVY0_BOVIN	Keratin	*	*
tr|Q17QG8|Q17QG8_BOVIN	Histone H2A	*	*

## 2 Experimental design, materials and methods

### 2.1 Biological material

The biological materials, metacercariae, were purchased from the laboratory DILAVE “Miguel C. Rubino” in Montevideo, Uruguay (Rubino strain) and from Baldwin Aquatics Inc. in Oregon, USA (US Pacific North West wild strain). The Rubino strain derived from cercariae developed in *Lymnaea viatrix*, and the US Pacific North West wild strain derived from cercariae developed in *Pseudosuccinea (Lymnaea) columella.* NEJ in vitro excystation proceeded at the same time and laboratory conditions for both strains. In brief, metacercariae (n = 500–1000) were activated in vitro and NEJ were allowed to excyst as previously described [4]. The emerging NEJ (n = 200–600) were washed with sterile phosphate-buffered saline (PBS, pH 7.4), and maintained at 37 °C, 5% CO_2_ in 1 mL of sterile culture medium (RPMI 1640, 30 mM HEPES pH 7.2, 2% glucose and 10% penicillin/streptomycin/amphotericin B mix). The ESP-containing supernatants were collected after incubation for 12 h. All ESP supernatant samples were syringe-filtered (0.22 μm), and concentrated using centrifugal filter of 3 kDa molecular weight cut-off. The protein concentrates were quantified by absorbance at 280 nm in a Nanodrop 1000 spectrophotometer (Thermo Fisher Scientific, USA), lyophilized, and stored at - 80 °C.

### 2.2 LC-MS-MS sample preparation

Lyophilized ESP samples were diluted in a buffer containing 8 M urea/0.1 M Tris, pH 8.5, reduced with 5 mM Tris (2-carboxyethyl) phosphine hydrochloride, and alkylated with 25 mM iodoaceamide. The ESP samples were hydrolyzed overnight at 37 °C in 2 M urea/0.1M Tris pH 8.5, 1 mM CaCl_2_ buffer with trypsin (final ratio 1:20, enzyme: substrate). Formic acid (5% final concentration) was added to stop the reaction, and samples were centrifuged at 17,000 g for 5 min at 4 °C for debris removal. Reversed-phase pre-columns were prepared in 250 μm ID/360 μm OD capillaries with a Kasil frit at one end. Pre-columns were packed in-house with 2 cm of a reversed-phase resin (5-μm ODS-AQ C18) particle slurry stored in methanol. Analytical reversed-phase columns were prepared by pulling a 100 μm ID/360 μm OD silica capillary into a 5-μm ID tip. Reversed-phase resin (20 cm) was packed directly onto the pulled column. Pre-columns and analytical columns were connected using a zero-dead volume union.

### 2.3 LC-MS/MS

Nanoflow LC-MS/MS with an Easy NanoLC II coupled to a Q Exactive mass spectrometer (Thermo Fisher Scientific, USA) was used to analyze the peptide mixtures. Mobile phases were solution A (5% acetonitrile/0.1% formic acid) and solution B (80% acetonitrile/0.1% formic acid), at a flow rate of 400 nL/min. Sample material (1.5 μg per injection) was applied into the column. LC-MS/MS was performed in 155-min chromatographic runs as follows: 1–10% B for 10 minutes, 10–40% B for 100 minutes, 40–50% B for 10 minutes, and 50–90% B in 10 minutes. The column was flushed with 90% B for 10 minutes, then brought back to 1% B, and re-equilibrated prior to the next injection. Peptides eluted from the analytical column were electrosprayed directly into the mass spectrometer.

The mass spectrometer was operated in a data-dependent mode, collecting a full MS scan from 400 to 1,200 m/z at 70,000 resolution and AGC target of 1 × 10^6^. The 10 most abundant ions in each MS scan were selected for MS/MS with AGC target of 2 × 10^5^, and an underfill ratio of 0.1%. Normalized collision energy was set to 25 and maximum fill times were 20 ms and 120 ms for MS and MS/MS scans, respectively, with dynamic exclusion of 15 s. Peptide and protein identification was done with Integrated Proteomics Pipeline–IP2 (Integrated Proteomics Applications, http://www.integratedproteomics.com). Tandem mass spectra were extracted from Thermo RAW files using RawExtract 1.9.9.2 [5], and searched with ProLuCID [6] against a non-redundant database comprising coding sequences from *Fasciola hepatica* genome [2] concatenated with a *Bos taurus* Uniprot reference database, in addition to reverse sequences of all entries. The search space included all fully-tryptic and half-tryptic peptide candidates with no missed cleavage restrictions. Carbamidomethylation on cysteine residues was used as a static modification. Data was searched with 50 ppm precursor ion tolerance and 20 ppm fragment ion tolerance. Identified proteins were filtered using DTASelect. Filtering required a minimum of 2 peptides per protein, at least one tryptic terminus for each peptide identification, and less than 1% FDR (false discovery rate). Normalized spectral abundance factor (NSAF) of the different samples was calculated according to Zybailov et al. [7]. A volcano plot was generated by pairwise comparison between ESP derived from NEJ from *L. viatrix* and from *P. columella* snails, using the PaternLab's TFold module [8].

### 2.4 Functional annotation

The functional annotation and classification of the matched proteins by BLASTP searches against several databases were performed using a program developed and provided by Dr. José M. C. Ribeiro [9]. After annotation, proteins for each dataset were manually curated or annotated, and results were compiled in hyperlinked Excel spreadsheets (Supplementary Table 1 and Supplementary Table 2), as well as a table comparing *B. taurus* proteins found in both samples (Table 1). Proteomic profiles were compared between samples as functional categories or individual proteins. For data comparison, we used 90 proteins identified in our previously published study with ESP from *F. hepatica* NEJ derived from *P. columella* metacercaria [10]. Both *F. hepatica* metacercariae batches were obtained, processed and analyzed at the same time.
